# Cellulose-based functional hydrogels derived from bamboo for product design

**DOI:** 10.3389/fpls.2022.958066

**Published:** 2022-08-16

**Authors:** Xiaobing Cao, Fei Li, Tingting Zheng, Guohui Li, Wenqian Wang, Yanjun Li, Siyu Chen, Xin Li, Yi Lu

**Affiliations:** ^1^School of Art and Design, Bamboo Research Institute, Zhejiang Provincial Collaborative Innovation Center for Bamboo Resources and High-Efficiency Utilization, Zhejiang A&F University, Hangzhou, China; ^2^School of Science and Technology, Huzhou College, Huzhou, China; ^3^School of Materials Engineering, Nanjing Forestry University, Nanjing, China; ^4^Department of Chemical and Biological Engineering, University of British Columbia, Vancouver, BC, Canada; ^5^Institute of Technical and Macromolecular Chemistry, RWTH Aachen University, Aachen, Germany; ^6^Institute of Biotechnology, RWTH Aachen University, Aachen, Germany

**Keywords:** bamboo cellulose, hydrogels, food packaging, environmental protection, plant agriculture, biomedicine

## Abstract

Hydrogels have outstanding research and application prospects in the field of product design. Among them, the design and preparation of cellulose-based functional hydrogels derived from bamboo have attracted increasing research interest. Cellulose-based hydrogels not only have the skeleton function of hydrogels, but also retain excellent specificity, smart structural design, precise molecular recognition ability, and superior biocompatibility. Cellulose-based hydrogels show important application prospects in various fields, such as environmental protection, biomedicine, and energy. What’s more, they are potentially viable for application in food packaging and plant agriculture, such as fertilizers release and crop production. Recently, researchers have extracted cellulose from bamboo and generated a variety of cellulose-based functional hydrogels with excellent properties by various cross-linking methods. In addition, a variety of multifunctional hybrid cellulose-based hydrogels have been constructed by introducing functional components or combining them with other functional materials, thus expanding the breadth and depth of their applications. Herein, we elaborate on advances in the field of cellulose-based hydrogels and highlight their applications in food packaging and plant agriculture. Meanwhile, the existing problems and prospects are summarized. The review provides a reference for the further development of cellulose-based hydrogels.

## Introduction

Bamboo is a kind of biomass material with a short growth cycle and excellent performance. Known as the “Kingdom of Bamboo,” China is the world’s richest country in bamboo resources (Kole et al., 2016; Yu H. L. et al., 2017). Owing to the excellent properties of biodegradability, low density, and superior mechanical strength, bamboo has been widely used in the reinforcement of polymers and functional composites production (Lin et al., 2021). Similar to other plants, such as wood, the chemical compositions of bamboo are cellulose, hemicellulose, and lignin, as well as other ingredients, such as sugars, fats, proteins, and inorganic salts (Marmiroli et al., 2005). In bamboo, cellulose accounts for 44% of the total bamboo, and lignin accounts for 20%. Bamboo mainly contains sclerenchyma fibers and parenchyma cells, which have different structures and compositions. Parenchyma cells make up 80% of bamboo processing residues produced in China every year, indicating that parenchyma cells are an excellent raw material for the preparation of nanocellulose (Ren et al., 2021, 2022). Compared with wood, the parenchyma of bamboo has thinner cell walls, larger microfibril angles, lower lignification, and easy peeling of wall layers, which facilitates cell wall dispersion (Cai et al., 2013; Lin et al., 2021; Li et al., 2022e).

**FIGURE 1:**
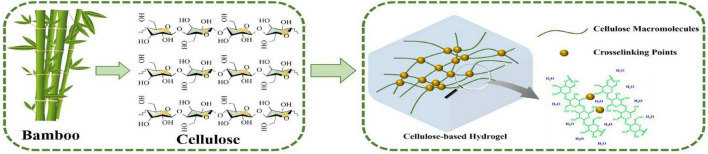
Schematic illustration of the structures of cellulose and cellulose-based hydrogels as well as the preparation process of cellulose-based hydrogels.

Various approaches have been used for the preparation of hydrogels from cellulose, indicating the huge application value of bamboo in the production of cellulose-based hydrogels because of the rich content of cellulose in bamboo. Cellulose is composed of β-D-glucopyranosyl (dehydrated glucose). The simple molecular formula is (C_6_H_10_O_5_)n. With crosslinking agents, cellulose can be used to produce cellulose-based hydrogels, which possess a three-dimensional network structure and modifiable physical and chemical properties (Figure 1). Cellulose-based hydrogels can be prepared by physical cross-linking of natural cellulose molecules or by chemical/physical cross-linking of cellulose derivatives with single or multiple process steps (Li et al., 2013; Shen et al., 2016). The single-step process typically includes polymerization techniques and parallel cross-linking of multiple monomers. The multiple steps include the synthesis of reactive groups of individual polymer molecules. Hydrogels can be designed and synthesized by scale control of a variety of hydrogel properties, such as structures, crosslink density, biodegradability, mechanical strength, chemical response, and hydrogel hydrology to stimuli (Li et al., 2016; Pin et al., 2016; Huber et al., 2019). In recent years, biomass resources have become impressive materials for hydrogel manufacturing due to their outstanding biodegradability and biocompatibility (Lahiani and Khodakovskaya, 2016). For example, cotton staple pulp has been used as a hydrogel material to form cellulose-based hydrogels through a single cross-linking agent (Li et al., 2017b,^2020^; Ye et al., 2019). Zhao et al. (2014) and Li et al. (2022a) prepared chitosan-based hydrogels by using chitosan extracted from chitin and dextran and applied hydrogels in drug delivery. Zheng et al. (2015) produced hydrogels using a molding and acidification process, and utilized generated hydrogels for self-healing applications as well as a sealant and gastric mucosa repair.

Due to the biodegradability, biocompatibility, non-toxicity, and functionality of cellulose, its derivatives have prompted scientists to explore their numerous applications (Navarra et al., 2015; Kole et al., 2016; Xie J. et al., 2016; Dutta et al., 2019; Li H. L et al., 2019; Li et al., 2022c). The low cost, lightweight, and biodegradability of cellulose-based hydrogel lead to its application in food packaging. With the hydrophilic property of hydrogels, cellulose hydrogels hold great promise for plant agriculture applications (Wei et al., 2022). Cellulose-based hydrogels are also considered useful biocompatible materials in medical devices (Yang et al., 2022). In addition, cellulose hydrogels have huge potential in applications for the environment, biomedicine, personal care products, and energy electronics.

In this review, the basic compositions of bamboo are first introduced. The extraction methods of cellulose or nanocellulose from bamboo and the strategies for preparing hydrogels with extracted cellulose are elaborated. In addition, the applications of cellulose-based hydrogels in various fields, such as food packaging, plant agriculture, environment, biomedicine, personal care products, and energy electronics are discussed. Finally, the future outlook of hydrogels in usage scenario and preparation technology is presented.

## Extraction of cellulose and nanocellulose from bamboo

Bamboo, an abundant lignocellulosic material with high cellulose content, has a strong potential to act as a biomass source for the production of cellulose and nanocellulose. Cellulose or nanocellulose extracted from bamboo has the characteristics of small environmental load, low weight, high adaptability, and relatively high strength (Cai et al., 2013). Generally speaking, the diameter of nanocellulose ranges from a few nanometers to tens of nanometers, and the length is more widely distributed. The nanocellulose in wood has a diameter of 3–5 nm and a length of 100–200 nm. The nanocellulose in seagrass has a diameter of about 20 nm and a length of 200–1,000 nm. The diameter of nanocellulose in bamboo is about 8 nm and the length is about 100 nm. There is a lot of literature on the extraction methods of cellulose and nanocellulose. In recent years, the development of extraction methods of cellulose and nanocellulose has been summarized in Table 1.

**TABLE 1:** Methods for extracting cellulose and nanocellulose.

Extracted materials	Extraction method	Evaluation of method	References
Cellulose	Cross Bevan method	Serious environmental pollution	Cross and Bevan, 1907
	Nitric acid ethanol hair	Low product extraction rate	Brendel et al., 2000; Khristova et al., 2002; Chen et al., 2011
	Alkali bleaching process	Good effect	Brienzo et al., 2009; Eliangela et al., 2011; Yang et al., 2013; Laurén et al., 2014; Kabir et al., 2018; Zainal et al., 2021; Bhaladhare and Das, 2022
Nanocellulose	Acid hydrolysis	Main preparation methods	Revol et al., 1992; Wang et al., 2008; Habibi et al., 2010
	Physical mechanical method	Environmentally friendly	Ahola et al., 2008; Dufresne et al., 2008; István and Plackett, 2010; Zhang et al., 2012
	Enzymolysis	Mild process conditions	Naeimi and Moradian, 2008
	Solvent method	Limited	Oksman et al., 2006; Sui et al., 2008; Liu et al., 2019

### Extraction method of bamboo cellulose

**FIGURE 2:**
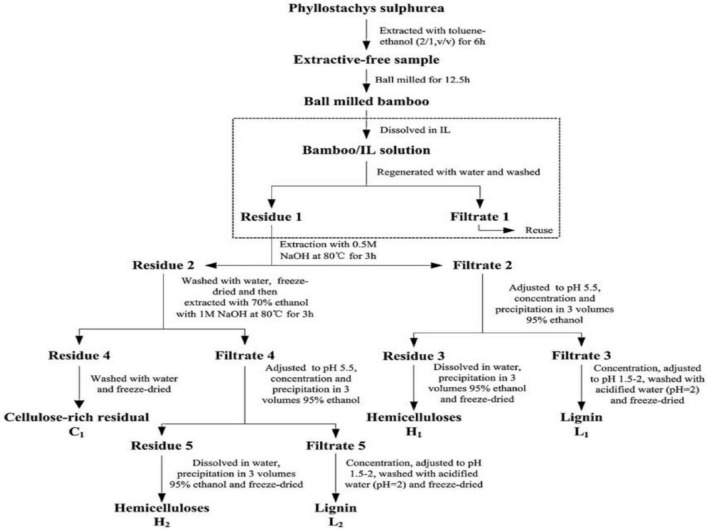
The process of extracting cellulose, hemicellulose, and lignin from bamboo with the assistance of ILs. Reprinted from Yang et al. (2013) with permission from ELSEVIER.

The bleach treatment-alkali treatment method is currently a relatively common method for extracting pure cellulose from moso bamboo (Laurén et al., 2014; Li et al., 2014a; Xie J. et al., 2016; Kabir et al., 2018; Zainal et al., 2021; Bhaladhare and Das, 2022). The method, consisting of a bleaching treatment step and an alkali treatment step, was employed to remove lignin and hemicellulose from moso bamboo materials, respectively, and therefore obtain cellulose with high purity. During the process of cellulose extraction, bleach with strong oxidizing property was utilized to remove lignin from moso bamboo materials. Afterward, a large amount of hemicellulose remains in the plant materials, and further removal of hemicellulose is required. Subsequently, under certain temperature conditions, lignin was removed from moso bamboo by immersing it in alkali solution to dissolute and degrade remaining hemicellulose. The bleaching oxidants in this method are mainly sodium chlorite, sodium hypochlorite, or chlorine, and the alkali reagents are mainly strong alkali reagents, such as lithium hydroxide, sodium hydroxide, and potassium hydroxide. Chen et al. (2011) treated the moso bamboo with acidic sodium chlorite solution under the pH of 4.0–5.0. The above operation was repeated six times to remove the lignin from the samples. The samples were then stirred for 2.0 h at 90°C with different concentrations of potassium hydroxide solution to remove hemicellulose, and finally chemically purified cellulose with high cellulose purity was obtained. This method can effectively remove the lignin and hemicellulose from moso bamboo materials. During the extraction process, the aggregation state and the physicochemical properties of cellulose were not affected significantly. As a result, the prepared purified cellulose can be widely used in nanocellulose materials production (Lu et al., 2021). Additionally, Yang et al. (2013) explored the potential application of green solvent ionic liquids (ILs) [Amim]Cl pretreatment on the extraction of cellulose from bamboo (Figure 2). As a result, increased accessibility of cellulose and partially fracture side-chains of hemicelluloses of [Amim]Cl were confirmed. In addition, the slight degradation of lignin and hemicelluloses fractions were observed during [Amim]Cl treatment.

### Extraction method of bamboo nanocellulose

Nanocellulose is a kind of natural, non-polluting, bio-tolerable, and environmentally friendly material. It possesses a special nano-size structure, excellent mechanical properties, biodegradable properties, and no rejection to biological organisms. The utilization of nanocellulose derived from bamboo is also an alternative to improve the values of bamboo residues and advance nanocellulose hydrogel development (Wang et al., 2015; Wang H. et al., 2016; Xie J. L. et al., 2016; Lu H. L. et al., 2018). To extract the nanocellulose from bamboo, various processes have been applied. For example, Liu et al. (2019) delignified moso bamboo and prepared nanocellulose by using a deep eutectic solvent (DES), which consisted of choline chloride (ChCl) and lactic acid (LC). With this method, the produced nanocellulose films show a high tensile strength within the range between 163 and 213 MPa. Recently, the combination of microwave liquefaction with a co-solvent dissolving system with dimethyl sulfoxide (DMSO) and tetrabutylammobium acetate (TBAA) has been developed to prepare nanocellulose from bamboo residues (Shao et al., 2021). As a result, the produced nanocellulose films exhibited good tensile strength (15–25 MPa) and displayed a homogeneous network structure. For the application, nanocellulose is mainly used in polymer matrix composites and plasticizer of cellulose materials, such as transparent nanocellulose films.

## Preparation of hydrogels with cellulose

The attractive properties of cellulose and its derivatives, such as biodegradability, biocompatibility, non-toxicity, usability, and functionality, have led worldwide scientists and researchers to develop cellulose-based hydrogels that can be used in a variety of applications (Zhou et al., 2015; Shen et al., 2016; Farag and Rostom, 2017; Li et al., 2017c; Ghorbani et al., 2018; Alven and Aderibigbe, 2020; Liu et al., 2020; Ji et al., 2021).

Cellulose-based hydrogels are generally prepared by physical crosslinking, chemical crosslinking, and polymerization technology (Table 2). A physical crosslinking method could be employed to improve hydrogels structures and mainly includes freeze-thawing technology (Zhang et al., 2013; Butylina et al., 2016; Timofejeva et al., 2017), photoinitiator technology (Lu M. et al., 2018; Qi et al., 2018; Yuan et al., 2018), and radiation induced technology (Singh and Bala, 2014; Gonzalez-Torres et al., 2018). For instance, Lu M. et al. (2018) employed photoinitiator technology to prepare glycol chitosan (GC) hydrogel. After illumination with blue light in the presence of ruthenium complex, the crosslinking of GC conjugated with phenolic groups was initiated. The produced hydrogels have outstanding tissue adhesiveness, which can be applied to wound healing.

**TABLE 2:** Methods for cellulose hydrogels preparation.

Preparation methods	Techniques/Agents	Advantages of produced hydrogels	References
Physical crosslinking	Freeze-thawing technique	Improved thermal stability, compressive strength, and crystallinity	Guan et al., 2014
	Photoinitiator technique	Good tissue adhesiveness, good hemostatic ability, and good anti-bacterial ability	Lu M. et al., 2018; Qi et al., 2018; Yuan et al., 2018
	Radiation-induced technique	Stability efficiency, high mechanical strength, and thermosensitive	Elbarbary et al., 2017; Barba et al., 2018
Chemical crosslinking	Citric acid (CA)	Improved water swelling, thermal stability, tensile strength, and barrier properties	Gyawali et al., 2010; Ghorpade et al., 2018
	Epichlorohydrin (ECH)	Enhanced pore size distribution, chemical stability, mechanical resistance, and adsorption/desorption capacity	Meybodi et al., 2013
	Glutaraldehyde (GA)	Improved biocompatibility, swelling behavior, more pH-sensitive, and increased hydrogel viscosity	Yu S. et al., 2017
Polymerization technique		Biodegradability and biocompatibility	Mohite and Adhav, 2017

A chemical crosslinking method is utilized to form the bonds between the polymer and crosslinking agents. In the chemical crosslinking method, many crosslinking agents, such as citric acid (CA) (Menzel et al., 2013; Wang et al., 2014; Seligra et al., 2016), epichlorohydrin (ECH) (Laus and Fávere, 2011; Jawad and Nawi, 2012), and glutaraldehyde (Dmitriev et al., 2015; Wang W. et al., 2016; Guoxin et al., 2017) were used. Yu S. et al. (2017) used glutaraldehyde (GA) as an agent to crosslink poloxamer 407 (F127) and carboxymethyl chitosan (CMC) and prepare hydrogels. The produced stimulus responsive three-dimensional cross-linked hydrogel system was composed of polyethene/polypropylene oxide/polyethylene oxide (PEO-PPO-PEO) block copolymer. The results showed that the hydrogel and its physical mixture had no cytotoxicity to human corneal epithelial cells at low concentration.

Additionally, a polymerization technique is also used for crosslinking in the preparation of hydrogel. Polymerization could be classified into three approaches, which are bulk polymerization, solution copolymerization, and polymerization by irradiation (Komatsu et al., 2019; Sc et al., 2020). Sc et al. (2020) prepared polyethylene glycol (PEG) hydrogels by free-radical polymerizations and investigated the effects of chondrocytes on hydrogels formation. Studies have shown that photopolymerized PEG hydrogels are a promising platform for chondrocyte encapsulation and cartilage tissue engineering.

## Applications of hydrogels

Nowadays, cellulose-based hydrogels have wide applications in food packaging, plant agriculture, environment, biomedicine, personal care products, and energy electronics due to their hydrophilicity, biodegradability, biocompatibility, non-toxicity, and remarkable solvent uptake (Table 3).

**TABLE 3:** Applications and characteristics of cellulose-based hydrogels.

Applications	Characteristics	References
Food packaging	Low cost, light weight, good mechanical property, high resistance, biodegradability,	Puligundla et al., 2012; Dai et al., 2021
Plant agriculture	High hydrophilic capacity	Bortolin et al., 2013; Ekebafe et al., 2013
Environment	Excellent adsorption property	Rigas et al., 1999; Huettermann et al., 2009; Ni et al., 2011; Mohammed et al., 2015
Biomedicine	Good mechanical properties, biocompatibility and renderability	Shanmugasundaram and Gowda, 2010; Erizal et al., 2014; Ma et al., 2015; Barleany et al., 2016; Onofrei and Filimon, 2016
Personal care products	Highly absorption	Lei et al., 2017; Li et al., 2017a; Liao et al., 2017; Si et al., 2017; Ge et al., 2018; Ge et al., 2021
Energy electronics	Mechanical flexibility	Lei et al., 2017; Li et al., 2017a; Liao et al., 2017; Si et al., 2017; Ge et al., 2018, 2021

### Hydrogels in food packaging

**FIGURE 3:**
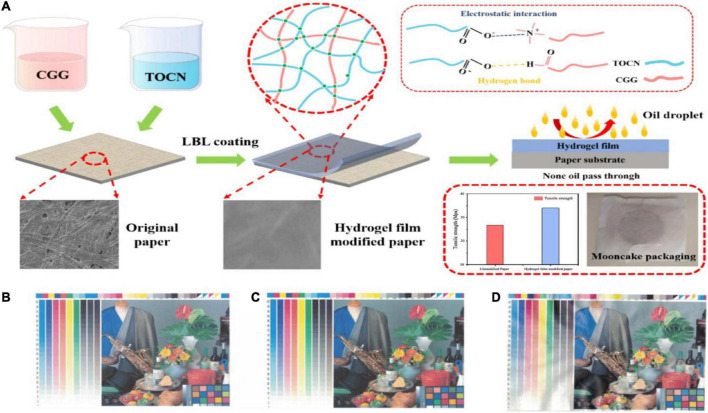
**(A)** Schematic illustration of the 2,2,6,6-tetramethylpiperidine-1-oxyl (TEMPO)-oxidized cellulose nanofiber/cationic guar gum (TOCN/CGG) self-assembled hydrogel film modified paper for food packaging. Inkjet printing effects on **(B)** ordinary printing paper, **(C)** unmodified paper, and **(D)** 4-layer hydrogel film modified paper. Reprinted from Dai et al. (2021) with permission from ELSEVIER.

Cellulose-based hydrogels have been widely used in food packaging due to their excellent properties, such as low cost, light weight, and good mechanical properties. In recent years, efforts have been made to explore alternatives to replace petroleum-based packaging materials to solve ecological problems, such as energy crisis and global warming. Cellulosic paper has received the widespread attention of researchers because of its low cost, light weight, and biodegradability. Dai et al. (2021) used 2,2,6,6-tetramethylpiperidine-1-oxyl (TEMPO)-oxidized cellulose nanofiber (TOCN)/cationic guar gum (CGG) hydrogel film to modify traditional cellulose paper and produce food packaging materials with good mechanical properties, barrier properties, and oil resistance (Figure 3). The results showed that compared with the unmodified paper, the tensile strength and elongation at break of the hydrogel film-modified paper increased by 13.4 and 27.1%. The water vapor transmission rate and the oil absorption rate decreased by 17.5 and 73.5%, respectively. In addition, after a period of time storage, the peroxide value of mooncake bags made from hydrogel film modified paper was still within the maximum value (0.25 g/100 g) specified by GB 7,099–2,015, which proved that the hydrogel film modified paper has good resistance to acid decay and provided new possibilities for the development of novel food packaging materials. The development of an intelligent food packaging material that integrates packaging, detection, and recording functions is of great interest. Additionally, the intelligent food packaging material can be used to monitor the freshness, maturity, and spoilage of food, mainly by reacting with microbial growth or a gas produced during food spoilage. CO_2_ is a common by-product of food spoilage process, and monitoring CO_2_ content in food is one of the common methods to measure freshness (Puligundla et al., 2012). The freshness of fruits, which is reflected by CO_2_ content, can be detected by the produced weakly acidic carbonic acid in the reaction of CO_2_ with water in the hydrogels.

### Hydrogels in plant agriculture

**FIGURE 4:**
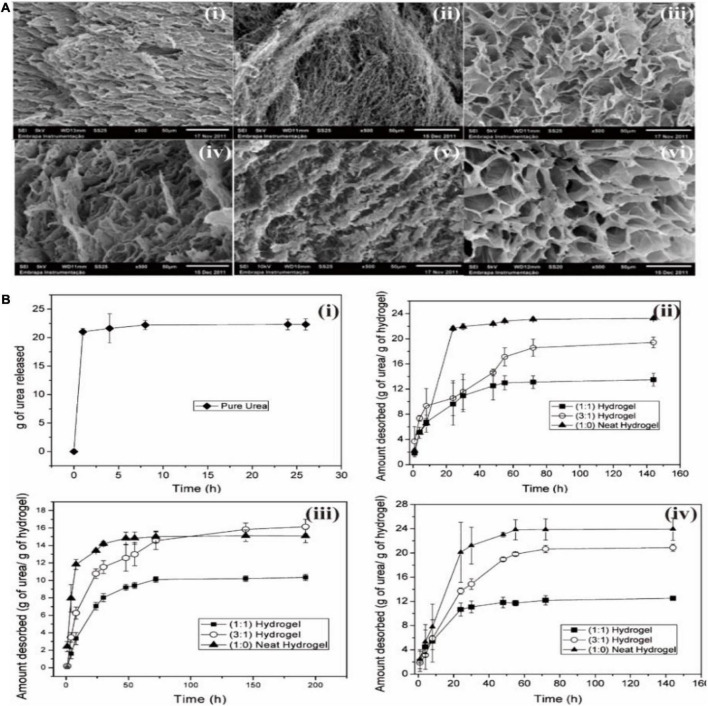
**(A)** Scanning electron microscopy (SEM) pictures of (i) (1:0) neat hydrogel; (ii) (1:0) hydrolyzed neat hydrogels; (iii) (1:1) hydrogel; (iv) (1:1) hydrolyzed hydrogel; (v) (3:1) hydrogel; and (vi) (3:1) hydrolyzed hydrogel. **(B)** The controlled desorption of urea for (i) pure spherical urea, and hydrogels added with different amount of clay mineral at (ii) pH 4.0, (iii) pH 7.0, and (iv) pH 9.0. Reprinted from Bortolin et al. (2013) with permission from ACS.

Hydrogels are receiving great attention in plant agriculture since hydrogels are extremely hydrophilic polymers. For instance, Bortolin et al. (2013) prepared hydrogels with polyacrylamide (PAAm), methyl cellulose (MC), and calcium montmorillonite (MMt). The produced hydrogels were utilized for the controlled release of fertilizers through the sorption and desorption studies of a nitrogenated fertilizer, urea [CO(NH_2_)_2_]. As shown in Figure 4A, the prepared hydrogels show quite homogeneous foliaceous structures. The pore morphology of hydrogels did not change significantly with the addition of clay. However, the pore size increased after the hydrolysis treatment. As a result, hydrogels show the controlled release of urea in different pHs (4,7, and 9) and the addition of clay mineral improved the controlled release of urea (Figure 4B). Ekebafe et al. (2013) prepared hydrogels from bamboo-based cellulose and other materials for seed culture applications. The produced hydrogels maintained the soil nutrient balance and improved the water holding capacity of the soil. It was found that this hydrogel resulted in a significant increase in the plant height, stem thickness, leaf area, biomass accumulation, relative fruit water content, and protein and sugar content.

### Hydrogels in environment

Cellulose-based hydrogels have been widely used to remove some impurities, such as heavy metal ions in the field of environmental protection because of their excellent adsorption. In the wastewater treatment, nanocellulose-based hydrogels are inexpensive, efficient, and recyclable adsorbent materials for the adsorption of heavy metal ions, dyes, and oily wastewater, etc. The high porosity and high specific surface area of cellulose nanofibril (CNF) aerogel make this material having excellent adsorption properties, and it has great potential as a high-performance oil absorption material in oil spill treatment. Mohammed et al. (2015) made Cap, n, collar (CNC)-sodium alginate (ALG) hydrogel from CNC and ALG with good adsorption and recyclability. Compared with pure ALG hydrogel, CNC-ALG hydrogel showed better adsorption of methylene blue (MB) with a maximum adsorption capacity of 256.4 mg/g, and the MB removal rate was still around 97% after five adsorption-desorption cycles. Materials controlled by hydrogel networks significantly reduce the frequency of agricultural irrigation, and film-coated fertilizers can reduce the environmental pollution (Rigas et al., 1999; Huettermann et al., 2009). Hydrogel-coated nitrogen fertilizer formulations based on carboxymethyl cellulose (CMC) and hydroxyethyl cellulose (HEC) were developed by Ni et al. (2011) for controlled and efficient release and to improve the water holding capacity of soils. In a related study, clay and herbicide (ethephon) were wrapped around a carboxymethylcellulose hydrogel, which allowed for the slow and controlled release of herbicide (Li et al., 2008).

### Hydrogels in biomedicine

In biomedicine, the three-dimensional (3D) network structure of nanocellulose-based hydrogels is similar to that of human tissues. Additionally, nanocellulose-based hydrogels have good mechanical properties, biocompatibility, and renderability, which makes them widely used in the fields of drug delivery, tissue engineering, trauma dressing, and wearable sensors (Li et al., 2014b, 2015, 2021a,b,c,d, 2022b; Li X. et al., 2019; Xing et al., 2020; Nik Nabil et al., 2022). Liu et al. (2018) added aminated silver nanoparticles (Ag-NH_2_NPs) and gelatin (G) to TOCNF. When Ag-NH_2_NPs were added with the concentration of 0.5 mg/ml, CNF/G/Ag hydrogel showed good mechanical properties, biocompatibility, and wound healing effect. After 14 days of treatment, the wound healing rate and survival rate were nearly 90 and 83.3%, respectively. Liu et al. (2016) prepared a composite hydrogel by the chemical modification of carboxymethyl fibers from bamboo shoot cellulose. Sodium salicylate was used as a model drug to study the adsorption and release behavior of the hydrogels in simulated intestinal (pH 7.4) and gastric juice (pH 1.8) environments. The release rate of the prepared composite hydrogels was higher in simulated intestinal fluid (63.09% after 380 min) than in gastric fluid (22.09% after 400 min). These pH responses of the prepared composite hydrogels, especially as drug carriers, show their potential application of controlled release of drugs in different environmental conditions or human organs. Tovar-Carrillo et al. (2013) prepared cellulose hydrogel membranes for cell culture scaffolds by using bamboo fibers as raw material. Three types of hydrogel membranes were described and their properties were compared to evaluate the effectiveness of the dissolution methods. The results indicated that the hydrogel membranes prepared with cellulose solution by the N-dimethylacetamide (DMAc)/LiCl method have good cytocompatibility for cell culture scaffolds. Hai et al. (2018) developed a ClO^–^ and SCN^–^ excited reversible responsive lanthanide luminescent Tb (III)-CMC complex hydrogel for selective detection, protection, and storage of fingerprint information. Compared with conventional fluorescent probes, the Tb (III)-CMC complex hydrogel can ensure the confidentiality of fingerprint information.

### Hydrogels in personal care products

Cellulose-based hydrogels have been widely used in the field of personal care products due to their excellent high absorption. Cellulose-based hydrogels are excellent alternatives for the development of highly absorbent, eco-friendly, and compostable materials for personal care products (Qureshi et al., 2020). Barleany et al. (2016) produced highly absorbent hydrogels with significant antimicrobial activity that can be applied in baby diapers and sanitary napkins. For hygiene product applications, highly absorbent materials with antimicrobial activity are needed to prevent skin irritation. The hydrogels synthesized by Erizal et al. (2014) through the radiation copolymerization reaction are fast absorbing and can be used in personal care and hygiene products, such as surgical pads, hot and cold therapy packs, medical waste curing, disposable diapers, and sanitary napkins. Shanmugasundaram and Gowda (2010) studied the application of hydrogels made from four different fiber compositions [pure bamboo, cotton, bamboo/cotton (70/30), and bamboo/cotton (50/50)] in infant diapers. The prepared diapers were characterized in terms of absorbency, liquid penetration, acquisition time under load, and rewetting of the diapers under load. The performance of bamboo/cotton (70/30) fiber blended diapers was found to be superior to other fiber blends. In addition, many promising applications were explored as a protective barrier for volatile organic compounds into the environment and as an absorbent for waste oil (Ma et al., 2015). Pittler and Ernst (2004) incorporated linen yarn waste into a highly absorbent hydrogel and produced a sanitary napkin product. As a result, the prepared sanitary napkin product has excellent biodegradability and higher water absorption property than currently marketed sanitary napkin products. Obtaining recyclable disposable diapers, napkins, and other sanitary products is one of the important goals of the modern industry. The use of fully biodegradable cellulose-based highly absorbent resins can be a good solution to these problems (Onofrei and Filimon, 2016).

### Hydrogels in energy electronics

Due to its excellent mechanical flexibility, cellulose-based hydrogels have been widely used in the field of energy electronics. At energy electronics level, Ge et al. (2021) applied polyacrylamide/cellulose nanofibrils/highly soluble salt containing highly hydrated Li^+^ ion (PAM/CNF/LiCl) hydrogels as electrolytes in a double layer supercapacitor. The capacitors exhibited good mechanical flexibility, low temperature stability (the hydrogel did not freeze with 50% LiCl concentration at −80°C), and cycling stability (96% specific capacitance retention after 10,000 cycles), which helped to compensate for the environmental sensitivity of conventional conductive hydrogels and provided a new idea for the normal operation of devices under extreme cold conditions. Smart wearable devices are a hot research topic due to their potential applications in health monitoring. Self-healing wearable devices can restore their structure and function after damage and enhance their durability, reliability as well as safety (Li et al., 2017a). As one kind of typical soft and flexible material, self-healing hydrogels have attracted great interest in the development of self-healing wearable devices for human motion detection due to their good viscoelasticity, electrical conductivity, and biocompatibility (Lei et al., 2017; Si et al., 2017; Ge et al., 2018; Li et al., 2022d). Due to the excellent self-adhesive properties, high strain sensitivity, remarkable electrical stability, and rapid self-healing ability of self-healing hydrogels, wearable strain sensors assembled from gels can attach directly to human skin and detect large movements, such as joint bending and stretching for various human motions. In addition, gel strain sensors can accurately detect and rapidly identify subtle movements, such as pulse and respiration, that help monitor an individual’s health in real time during athletic training (Liao et al., 2017). This gel with high strain sensitivity is an ideal candidate for assembling scalable and wearable strain sensors in the application of human activity monitoring and personal medical diagnostics.

## Summary

This article mainly studies the preparation and application of bamboo-based cellulose hydrogels. Bamboo-based cellulose hydrogels can be used in food packaging, plant agriculture, environment, biomedicine, personal care products, and energy electronics. Compared with other wood, bamboo has many advantages, such as short growth cycle, low cost, and easy access to raw materials. Although cellulose-based composites have obvious advantages over pure cellulose-based composites and wide applications in the field of fillers, reinforcing agents, and stabilizers, their applications in biomedical engineering, food packaging, and cosmetics still need to be further expanded. Additionally, it is necessary to further investigate the properties of lignin in lignin nanofibers and its mechanism of action with the aim of fully developing the potential value of lignin cellulose materials and applications in various fields. Therefore, the future improvement of the preparation and application of cellulose-based hydrogels can be considered from the following aspects: (1) prepare cellulose-based hydrogels by combining cellulose and its derivatives with excellent properties. It is needed to optimize the preparation method, reduce the cost, and realize the transition from laboratory to industrialization as soon as possible. (2) Introduce more specific functional groups in the surface of cellulose, increase the cross-linking sites on the surface of cellulose, and thus improve the adsorption capacity of cellulose-based hydrogels for pollutants. (3) To promote the rapid development of bionic electronic devices, develop cellulose-based hydrogel sensors with good stretchability, frost resistance, adhesion, and self-healing properties. (4) Develop a smart fluorescent composite hydrogel with tunable luminescence properties and no irritant residue, and use it effectively for sensing detection, information storage and encryption, and water exploration and camouflage. This research can lay a good exploration foundation for the functionalization and high value-added application of bamboo.

## Author contributions

XC, XL, and YL conceptualized the manuscript. XC, FL, and GL wrote the draft manuscript. FL, TZ, WW, YLi, and SC modified the manuscript. XL and YLu reviewed and revised the manuscript. All authors approved for the final submitted version.

## References

[B1] Ahola S. Salmi J. Johansson L. S. Laine J. Osterberg M. (2008). Model films from native cellulose nanofibrils. Preparation, swelling, and surface interactions. *Biomacromolecules* 9 1273–1282. 10.1021/bm701317k 18307305

[B2] Alven S. Aderibigbe B. A. (2020). Chitosan and cellulose-based hydrogels for wound management. *Int. J. Mol. Sci.* 21:9656. 10.3390/ijms21249656 33352826 PMC7767230

[B3] Barba B. D. Aranilla C. T. Relleve L. S. Cruz V. R. C. Vista J. R. Abad L. V. (2018). Hemostatic granules and dressing prepared from formulations of carboxymethyl cellulose, kappa-carrageenan and polyethylene oxide crosslinked by gamma radiation. *Radiat. Phys. Chem.* 144 180–188. 10.1016/j.radphyschem.2017.08.009

[B4] Barleany D. R. Alim I. P. Rizkiyah N. Lusi U. T. Heriyanto H. Erizal E. (2016). “Chitosan-Graft-Poly (Acrylic Acid) superabsorbent hydrogel with antimicrobial activity,” in *Proceedings of the The First International Conference on Technology, Innovation, and Society*, West Sumatra, Indonesia, 654–661. 10.21063/ICTIS.2016.1099

[B5] Bhaladhare S. Das D. (2022). Cellulose: a fascinating biopolymer for hydrogel synthesis. *J. Mater. Chem. B* 10 1923–1945. 10.1039/D1TB02848K 35226030

[B6] Bortolin A. Aouada F. A. Mattoso L. H. Ribeiro C. (2013). Nanocomposite PAAm/methyl cellulose/montmorillonite hydrogel: evidence of synergistic effects for the slow release of fertilizers. *J. Agric. Food Chem.* 61 7431–7439. 10.1021/jf401273n 23822729

[B7] Brendel O. Iannetta P. Stewart D. (2000). A rapid and simple method to isolate pure alpha-cellulose. *Phytochem. Anal.* 11 7–10. 10.1002/(SICI)1099-1565(200001/02)11:1<7::AID-PCA488>3.0.CO;2-U

[B8] Brienzo M. Siqueira A. F. Milagres A. (2009). Search for optimum conditions of sugarcane bagasse hemicellulose extraction. *Biochem. Eng. J.* 46 199–204. 10.1016/j.bej.2009.05.012

[B9] Butylina S. Geng S. Oksman K. (2016). Properties of as-prepared and freeze-dried hydrogels made from poly(vinyl alcohol) and cellulose nanocrystals using freeze-thaw technique. *Eur. Polym. J.* 81 386–396. 10.1016/j.eurpolymj.2016.06.028

[B10] Cai J. Fei P. Xiong Z. Shi Y. Yan K. Xiong H. (2013). Surface acetylation of bamboo cellulose: preparation and rheological properties. *Carbohydr. Polym.* 92 11–18. 10.1016/j.carbpol.2012.09.059 23218259

[B11] Chen W. Yu H. Liu Y. Hai Y. Zhang M. Chen P. (2011). Isolation and characterization of cellulose nanofibers from four plant cellulose fibers using a chemical-ultrasonic process. *Cellulose* 18 433–442. 10.1007/s10570-011-9497-z

[B12] Dai L. Xi X. Li X. Li W. Du Y. Lv Y. (2021). Self-assembled all-polysaccharide hydrogel film for versatile paper-based food packaging. *Carbohydr. Polym.* 271:8425. 10.1016/j.carbpol.2021.118425 34364566

[B13] Dmitriev I. Kuryndin I. Bobrova N. Smirnov M. (2015). Swelling behavior and network characterization of hydrogels from linear polyacrylamide crosslinked with glutaraldehyde. *Mater. Today Commun.* 4 93–100. 10.1016/j.mtcomm.2015.06.005

[B14] Dufresne A. Habibi Y. Goffin A. L. Schiltz N. Duquesne E. Dubois P. (2008). Bionanocomposites based on poly(e-caprolactone)-grafted cellulose nanocrystals by ring opening polymerization. *J. Mater. Chem.* 18 5002–5010. 10.1039/b809212e

[B15] Dutta S. D. Patel D. K. Lim K. T. (2019). Functional cellulose-based hydrogels as extracellular matrices for tissue engineering. *J. Biol. Eng.* 13 1–19. 10.1186/s13036-019-0177-0 31249615 PMC6585131

[B16] Ekebafe L. Idiaghe J. Ekebafe M. (2013). Effect of delignified Native Bamboo (*Bambusa vulgaris*) Cellulosic–g-poly (*acrylonitrile*) hydrogel on the growth indices of Okra (*Abelmoschus esculentus*) seedlings. *Caspian J. Appl. Sci. Res.* 2 67–75.

[B17] Elbarbary A. M. Abd El-Rehim H. A. El-Sawy N. M. Hegazy E. S. A. Soliman E. S. A. (2017). Radiation induced crosslinking of polyacrylamide incorporated low molecular weights natural polymers for possible use in the agricultural applications. *Carbohydr. Polym.* 176 19–28. 10.1016/j.carbpol.2017.08.050 28927598

[B18] Eliangela T. Bondancia T. Teodoro K. Corrêa A. Marconcini J. Mattoso L. (2011). Sugarcane bagasse whiskers: extraction and characterizations. *Ind. Crops Prod.* 33 63–66. 10.1016/j.indcrop.2010.08.009

[B19] Erizal E. Perkasa D. P. Abbas B. Sudirman S. Sulistioso G. (2014). Fast swelling superabsorbent hydrogels starch based prepared by gamma radiation techniques. *Indones. J. Chem.* 14 246–252. 10.22146/ijc.21235

[B20] Cross C. F Bevan E. J. (1907). *Researches On Cellulose.* London: Longmann Green.

[B21] Farag R. K. Rostom M. (2017). Antimicrobial activity of carboxymethyl cellulose based nanogels. *Res. J. Pharm. Biol. Chem. Sci.* 8 2240–2251.

[B22] Ge G. Zhang Y. Shao J. Wang W. Si W. Huang W. (2018). Stretchable, transparent, and self−patterned hydrogel−based pressure sensor for human motions detection. *Adv. Funct. Mater.* 28:1802576. 10.1002/adfm.201802576 34842414

[B23] Ge W. Cao S. Yang Y. Rojas O. J. Wang X. (2021). Nanocellulose/LiCl systems enable conductive and stretchable electrolyte hydrogels with tolerance to dehydration and extreme cold conditions. *Chem. Eng. J.* 408:127306. 10.1016/j.cej.2020.127306

[B24] Ghorbani S. Eyni H. Bazaz S. R. Nazari H. Asl L. S. Zaferani H. (2018). Hydrogels based on cellulose and its derivatives: applications, synthesis, and characteristics. *Polym. Sci. A* 60 707–722. 10.1134/S0965545X18060044

[B25] Ghorpade V. S. Yadav A. V. Dias R. J. Mali K. K. Pargaonkar S. S. Shinde P. V. (2018). Citric acid crosslinked carboxymethylcellulose-poly(ethylene glycol) hydrogel films for delivery of poorly soluble drugs. *Int. J. Biol. Macromol.* 118 783–791. 10.1016/j.ijbiomac.2018.06.142 29959996

[B26] Gonzalez-Torres M. Leyva-Gomez G. Rivera M. Krotzsch E. Rodriguez-Talavera R. Leonor Rivera A. (2018). Biological activity of radiation-induced collagenpolyvinylpyrrolidonePEG hydrogels. *Mater. Lett.* 214 224–227. 10.1016/j.matlet.2017.12.006

[B27] Guan Y. Bian J. Peng F. Zhang X.-M. Sun R.-C. (2014). High strength of hemicelluloses based hydrogels by freeze/thaw technique. *Carbohydr. Polym.* 101 272–280. 10.1016/j.carbpol.2013.08.085 24299774

[B28] Guoxin T. Xinggang Y. Xiaoyu Z. Lei T. Yaxuan L. (2017). A novel pH-induced thermosensitive hydrogel composed of carboxymethyl chitosan and poloxamer cross-linked by glutaraldehyde for ophthalmic drug delivery. *Carbohydr. Polym.* 155 208–217. 27702506 10.1016/j.carbpol.2016.08.073

[B29] Gyawali D. Nair P. Zhang Y. Tran R. T. Zhang C. Samchukov M. (2010). Citric acid-derived in situ crosslinkable biodegradable polymers for cell delivery. *Biomaterials* 31 9092–9105. 10.1016/j.biomaterials.2010.08.022 20800893 PMC2954112

[B30] Habibi Y. Lucia L. A. Rojas O. J. (2010). Cellulose nanocrystals: chemistry, self-assembly, and applications. *Chem. Rev.* 110 3479–3500. 10.1021/cr900339w 20201500

[B31] Hai J. Li T. Su J. Liu W. Ju Y. Wang B. (2018). Reversible response of luminescent Terbium (III)–nanocellulose hydrogels to anions for latent fingerprint detection and encryption. *Angew. Chem. Int. Ed.* 57 6786–6790. 10.1002/anie.201800119 29660210

[B32] Huber T. Feast S. Dimartino S. Cen W. Fee C. (2019). Analysis of the effect of processing conditions on physical properties of thermally set cellulose hydrogels. *Materials* 12:1066. 10.3390/ma12071066 30939751 PMC6479291

[B33] Huettermann A. Orikiriza L. J. Agaba H. (2009). Application of superabsorbent polymers for improving the ecological chemistry of degraded or polluted lands. *Clean Soil Air Water* 37 517–526. 10.1002/clen.200900048

[B34] István S. Plackett D. (2010). Microfibrillated cellulose and new nanocomposite materials: a review. *Cellulose* 17 459–494. 10.1007/s10570-010-9405-y

[B35] Jawad A. H. Nawi M. A. (2012). Oxidation of crosslinked chitosan-epichlorohydrine film and its application with TiO2 for phenol removal. *Carbohydr. Polym.* 90 87–94. 10.1016/j.carbpol.2012.04.066 24751014

[B36] Ji L. Zhang F. Zhu L. Jiang J. (2021). An in-situ fabrication of bamboo bacterial cellulose/sodium alginate nanocomposite hydrogels as carrier materials for controlled protein drug delivery. *Int. J. Biol. Macromol.* 170 459–468. 10.1016/j.ijbiomac.2020.12.139 33359254

[B37] Kabir S. Sikdar P. P. Haque B. Bhuiyan M. Ali A. Islam M. (2018). Cellulose-based hydrogel materials: chemistry, properties and their prospective applications. *Prog. Biomater.* 7 153–174. 10.1007/s40204-018-0095-0 30182344 PMC6173681

[B38] Khristova P. Kordachia O. Patt R. Khider T. Karrar I. (2002). Alkaline pulping with additives of kenaf from Sudan. *Ind. Crops Prod.* 15 229–235. 10.1016/S0926-6690(01)00118-2 15364084

[B39] Kole C. Kumar D. S. Khodakovskaya M. V. (2016). *Plant Nanotechnology: Principles and Practices.* Berlin: Springer. 10.1007/978-3-319-42154-4

[B40] Komatsu S. Asoh T.-A. Ishihara R. Kikuchi A. (2019). Fabrication of thermoresponsive degradable hydrogel made by radical polymerization of 2-methylene-1,3-dioxepane: unique thermal coacervation in hydrogel. *Polymer* 179:121633. 10.1016/j.polymer.2019.121633

[B41] Lahiani M. H. Khodakovskaya M. V. (2016). Concerns about nanoparticle hazard to human health and environment. *Plant Nanotechnol.* 2016 349–365. 10.1007/978-3-319-42154-4_14

[B42] Laurén P. Lou Y.-R. Raki M. Urtti A. Bergström K. Yliperttula M. (2014). Technetium-99m-labeled nanofibrillar cellulose hydrogel for in vivo drug release. *Eur. J. Pharm. Sci.* 65 79–88. 10.1016/j.ejps.2014.09.013 25245005

[B43] Laus R. Fávere V. T. D. (2011). Competitive adsorption of Cu(II) and Cd(II) ions by chitosan crosslinked with epichlorohydrin-triphosphate. *Bioresour. Technol.* 102 8769–8776. 10.1016/j.biortech.2011.07.057 21824768

[B44] Lei Z. Wang Q. Sun S. Zhu W. Wu P. (2017). A bioinspired mineral hydrogel as a self−healable, mechanically adaptable ionic skin for highly sensitive pressure sensing. *Adv. Mater.* 29:1700321. 10.1002/adma.201700321 28417600

[B45] Li H. L. Li X. Jain P. Peng H. Rahimi K. Singh S. (2020). Dual-degradable biohybrid microgels by direct cross-linking of chitosan and dextran using azide-alkyne cycloaddition. *Biomacromolecules* 21 4933–4944. 10.1021/acs.biomac.0c01158 33210916

[B46] Li H. L. Mergel O. Jain P. Li X. Peng H. Rahimi K. (2019). Electroactive and degradable supramolecular microgels. *Soft Matter.* 15 8589–8602. 10.1039/C9SM01390C 31642835

[B47] Li J. Li Y. Dong H. (2008). Controlled release of herbicide acetochlor from clay/carboxylmethylcellulose gel formulations. *J. Agric. Food Chem.* 56 1336–1342. 10.1021/jf072530l 18232635

[B48] Li X. Lu S. Y. Xiong Z. G. Hu Y. Ma D. Lou W. Q. (2019). Light-Addressable nanoclusters of ultrasmall iron oxide nanoparticles for enhanced and dynamic magnetic resonance imaging of arthritis. *Adv. Sci.* 6:9. 10.1002/advs.201901800 31592427 PMC6774037

[B49] Li H. L. Wu X. Y. Li X. Cao X. B. Li Y. J. Cao H. R. (2021a). Multistage extraction of star anise and black pepper derivatives for antibacterial, antioxidant, and anticancer activity. *Front. Chem.* 9:14. 10.3389/fchem.2021.660138 34055736 PMC8160366

[B50] Li X. Li H. Zhang C. Pich A. Xing L. Shi X. (2021b). Intelligent nanogels with self-adaptive responsiveness for improved tumor drug delivery and augmented chemotherapy. *Bioact. Mater.* 6 3473–3484. 10.1016/j.bioactmat.2021.03.021 33869898 PMC8024537

[B51] Li X. Ouyang Z. Li H. Hu C. Saha P. Xing L. (2021c). Dendrimer-decorated nanogels: efficient nanocarriers for biodistribution in vivo and chemotherapy of ovarian carcinoma. *Bioact. Mater.* 6 3244–3253. 10.1016/j.bioactmat.2021.02.031 33778202 PMC7970313

[B52] Li X. Sun H. Li H. Hu C. Luo Y. Shi X. (2021d). Multi−Responsive biodegradable cationic nanogels for highly efficient treatment of tumors. *Adv. Funct. Mater.* 31:2100227. 10.1002/adfm.202100227

[B53] Li X. Xing L. Hu Y. Xiong Z. Wang R. Xu X. (2017b). An RGD-modified hollow silica@Au core/shell nanoplatform for tumor combination therapy. *Acta Biomater.* 62 273–283. 10.1016/j.actbio.2017.08.024 28823719

[B54] Li X. Xing L. X. Zheng K. L. Wei P. Du L. F. Shen M. W. (2017c). Formation of gold nanostar-coated hollow mesoporous silica for tumor multimodality imaging and photothermal therapy. *ACS Appl. Mater. Interfaces* 9 5817–5827. 10.1021/acsami.6b15185 28118704

[B55] Li J. Geng L. Wang G. Chu H. Wei H. (2017a). Self-healable gels for use in wearable devices. *Chem. Mater.* 29 8932–8952. 10.1021/acs.chemmater.7b02895

[B56] Li X. Xiong Z. Xu X. Luo Y. Peng C. Shen M. (2016). 99mTc-Labeled multifunctional low-generation dendrimer-entrapped gold nanoparticles for targeted SPECT/CT dual-mode imaging of tumors. *ACS Appl. Mater. Interfaces* 8 19883–19891. 10.1021/acsami.6b04827 27434031

[B57] Li X. Yang M. Shi X. Chu X. Chen L. Wu Q. (2015). Effect of the intramolecular hydrogen bond on the spectral and optical properties in chitosan oligosaccharide. *Physica E Low Dimens. Syst. Nanostruct.* 69 237–242. 10.1016/j.physe.2015.01.043

[B58] Li X. Yang M. S. Ye Z. P. Chen L. Xu C. Chu X. X. (2013). DFT research on the IR spectrum of glycine tryptophan oligopeptides chain. *Acta Phys. Sin.* 62:7. 10.7498/aps.62.156103

[B59] Li X. Yu S. Yang M. S. Xu C. Wang Y. Chen L. (2014a). Electronic structure analysis of glycine oligopeptides and glycine-tryptophan oligopeptides. *Physica E Low Dimens. Syst. Nanostruct.* 57 63–68. 10.1016/j.physe.2013.10.028

[B60] Li X. Zhang L. Yang M. Chu X. Xu C. Chen L. (2014b). Theoretical study on geometry and physical and chemical properties of oligochitosan. *Acta Phys. Sin.* 63:076102. 10.7498/aps.63.076102

[B61] Li Z. H. Chen C. J. Xie H. Yao Y. Zhang X. Brozena A. (2022e). Sustainable high-strength macrofibres extracted from natural bamboo. *Nat. Sustain.* 5:235. 10.1038/s41893-021-00831-2

[B62] Li H. Wei W. Xu H. (2022a). Drug discovery is an eternal challenge for the biomedical sciences. *Acta Mater. Medica.* 1 1–3. 10.15212/AMM-2022-1001

[B63] Li X. Kong L. D. Hu W. Zhang C. C. Pich A. Shi X. Y. (2022c). Safe and efficient 2D molybdenum disulfide platform for cooperative imaging-guided photothermal-selective chemotherapy: a preclinical study. *J. Adv. Res.* 37 255–266. 10.1016/j.jare.2021.08.004 35499043 PMC9039738

[B64] Li X. Hetjens L. Wolter N. Li H. Shi X. Pich A. (2022b). Charge-reversible and biodegradable chitosan-based microgels for lysozyme-triggered release of vancomycin. *J. Adv. Res.* 10.1016/j.jare.2022.02.014 PMC9811367 36585117

[B65] Li X. Lu Y. Hu Y. (2022d). A wireless and battery-free DNA hydrogel biosensor for wound infection monitoring. *Matter.* 10.1016/j.matt.2022.06.021

[B66] Liao M. Wan P. Wen J. Gong M. Wu X. Wang Y. (2017). Wearable, healable, and adhesive epidermal sensors assembled from mussel−inspired conductive hybrid hydrogel framework. *Adv. Funct. Mater.* 27:1703852. 10.1002/adfm.201703852

[B67] Lin Q. Q. Huang Y. X. Yu W. J. (2021). Effects of extraction methods on morphology, structure and properties of bamboo cellulose. *Ind. Crops Prod.* 169:7. 10.1016/j.indcrop.2021.113640

[B68] Liu H. Liu K. Han X. Xie H. Si C. Liu W. (2020). Cellulose nanofibrils-based hydrogels for biomedical applications: progresses and challenges. *Curr. Med. Chem.* 27 4622–4646. 10.2174/0929867327666200303102859 32124687

[B69] Liu Q. Yuan T. Fu Q. J. Bai Y. Y. Peng F. Yao C. L. (2019). Choline chloride-lactic acid deep eutectic solvent for delignification and nanocellulose production of moso bamboo. *Cellulose* 26 9447–9462. 10.1007/s10570-019-02726-0

[B70] Liu R. Dai L. Si C. Zeng Z. (2018). Antibacterial and hemostatic hydrogel via nanocomposite from cellulose nanofibers. *Carbohydr. Polym.* 195 63–70. 10.1016/j.carbpol.2018.04.085 29805020

[B71] Liu S. Luo W. Huang H. (2016). Characterization and behavior of composite hydrogel prepared from bamboo shoot cellulose and β-cyclodextrin. *Int. J. Biol. Macromol.* 89 527–534. 10.1016/j.ijbiomac.2016.05.023 27174909

[B72] Lu H. L. Zhang L. L. Liu C. C. He Z. B. Zhou X. F. Ni Y. H. (2018). A novel method to prepare lignocellulose nanofibrils directly from bamboo chips. *Cellulose* 25 7043–7051. 10.1007/s10570-018-2067-x

[B73] Lu L. Zou S. Fang B. (2021). The critical impacts of ligands on heterogeneous nanocatalysis: a review. *ACS Catal.* 11 6020–6058. 10.1021/acscatal.1c00903

[B74] Lu M. Liu Y. Huang Y. C. Huang C. J. Tsai W. B. (2018). Fabrication of photo-crosslinkable glycol chitosan hydrogel as a tissue adhesive. *Carbohydr. Polym.* 181 668–674. 10.1016/j.carbpol.2017.11.097 29254021

[B75] Ma J. Li X. Bao Y. (2015). Advances in cellulose-based superabsorbent hydrogels. *RSC Adv.* 5 59745–59757. 10.1039/C5RA08522E

[B76] Marmiroli M. Antonioli G. Maestri E. Marmiroli N. (2005). Evidence of the involvement of plant ligno-cellulosic structure in the sequestration of Pb: an X-ray spectroscopy-based analysis. *Environ. Pollut.* 134 217–227. 10.1016/j.envpol.2004.08.004 15589649

[B77] Menzel C. Olsson E. Plivelic T. S. Andersson R. Johansson C. Kuktaite R. (2013). Molecular structure of citric acid cross-linked starch films. *Carbohydr. Polym.* 96 270–276. 10.1016/j.carbpol.2013.03.044 23688480

[B78] Meybodi Z. E. Imani M. Atai M. (2013). Kinetics of dextran crosslinking by epichlorohydrin: a rheometry and equilibrium swelling study. *Carbohydr. Polym.* 92 1792–1798. 10.1016/j.carbpol.2012.11.030 23399221

[B79] Mohammed N. Grishkewich N. Berry R. M. Tam K. C. (2015). Cellulose nanocrystal–alginate hydrogel beads as novel adsorbents for organic dyes in aqueous solutions. *Cellulose* 22 3725–3738. 10.1007/s10570-015-0747-3

[B80] Mohite P. B. Adhav S. (2017). A hydrogels: methods of preparation and applications. *Int. J. Adv. Pharm.* 6 79–85. 34896566

[B81] Naeimi H. Moradian M. (2008). Alumina-supported metal(II) Schiff base complexes as heterogeneous catalysts in the high-regioselective cleavage of epoxides to halohydrins by using elemental halogen. *Polyhedron* 27 3639–3645. 10.1016/j.poly.2008.08.015

[B82] Navarra M. A. Dal Bosco C. Serra Moreno J. Vitucci F. M. Paolone A. Panero S. (2015). Synthesis and characterization of cellulose-based hydrogels to be used as gel electrolytes. *Membranes* 5 810–823. 10.3390/membranes5040810 26633528 PMC4704013

[B83] Ni B. Liu M. Lu S. Xie L. Wang Y. (2011). Environmentally friendly slow-release nitrogen fertilizer. *J. Agric. Food Chem.* 59 10169–10175. 10.1021/jf202131z 21848295

[B84] Nik Nabil W. N. Xi Z. Liu M. Li Y. Yao M. Liu T. (2022). Advances in therapeutic agents targeting quiescent cancer cells. *Acta Mater. Med.* 1 56–71. 10.15212/AMM-2021-0005

[B85] Oksman K. Mathew A. P. Bondeson D. (2006). Manufacturing process of cellulose whiskers/polylactic acid nanocomposites. *Compos Sci. Technol.* 66 2776–2784. 10.1016/j.compscitech.2006.03.002

[B86] Onofrei M. Filimon A. (2016). “Cellulose-based hydrogels: designing concepts, properties, and perspectives for biomedical and environmental applications,” in *Polymer Science: Research Advances, Practical Applications And Educational Aspects*, eds Méndez-Vilas A. Solano A. (Spain: Formatex Research Center), 108–120.

[B87] Pin L. W. Ashri A. Bakar M. A. Wan Y. Lazim M. (2016). Penyediaan dan pencirian hidrogel berasaskan kanji/akrilamida daripada UBI *Stemona Curtisii*. *Malays. J. Anal. Sci.* 20 157–170. 10.17576/mjas-2016-2001-17

[B88] Pittler M. H. Ernst E. (2004). Dietary supplements for body-weight reduction: a systematic review. *Am. J. Clin. Nutr.* 79 529–536. 10.1093/ajcn/79.4.529 15051593

[B89] Puligundla P. Jung J. Ko S. (2012). Carbon dioxide sensors for intelligent food packaging applications. *Food Control* 25 328–333. 10.1016/j.foodcont.2011.10.043

[B90] Qi C. Liu J. Jin Y. Xu L. Wang G. Wang Z. (2018). Photo-crosslinkable, injectable sericin hydrogel as 3D biomimetic extracellular matrix for minimally invasive repairing cartilage. *Biomaterials* 163 89–104. 10.1016/j.biomaterials.2018.02.016 29455069

[B91] Qureshi M. A. Nishat N. Jadoun S. Ansari M. Z. (2020). Polysaccharide based superabsorbent hydrogels and their methods of synthesis: a review. *Carbohydr. Polym. Technol. Appl.* 1:100014. 10.1016/j.carpta.2020.100014

[B92] Ren W. T. Guo F. Zhu J. W. Cao M. D. Wang H. K. Yu Y. (2021). A comparative study on the crystalline structure of cellulose isolated from bamboo fibers and parenchyma cells. *Cellulose* 28 5993–6005. 10.1007/s10570-021-03892-w

[B93] Ren W. T. Zhu J. W. Guo F. Guo J. Wang H. K. Yu Y. (2022). Estimating cellulose microfibril orientation in the cell wall sublayers of bamboo through dimensional analysis of microfibril aggregates. *Ind. Crops Prod.* 179:10. 10.1016/j.indcrop.2022.114677

[B94] Revol J. F. Bradford H. Giasson J. Marchessault R. H. Gray D. G. (1992). Helicoidal self-ordering of cellulose microfibrils in aqueous suspension. *Int. J. Biol. Macromol.* 14 170–172. 10.1016/S0141-8130(05)80008-X 1390450

[B95] Rigas F. Sachini E. Chatzoudis G. Kanellopoulos N. (1999). Effects of a polymeric soil conditioner on the early growth of sunflowers. *Can J. Soil Sci.* 79 225–231. 10.4141/S98-017

[B96] Sc A. Mmm A. Sjbab C. (2020). Cell encapsulation spatially alters crosslink density of poly(ethylene glycol) hydrogels formed from free-radical polymerizations - ScienceDirect. *Acta Biomater.* 109 37–50. 10.1016/j.actbio.2020.03.033 32268243 PMC7649065

[B97] Seligra P. G. Jaramillo C. M. Famá L. Goyanes S. (2016). Biodegradable and non-retrogradable eco-films based on starch-glycerol with citric acid as crosslinking agent. *Carbohydr. Polym.* 138 66–74. 10.1016/j.carbpol.2015.11.041 26794739

[B98] Shanmugasundaram O. Gowda R. (2010). Development and characterization of bamboo and organic cotton fibre blended baby diapers. *Indian J. Fibre Text. Res.* 35 201–205.

[B99] Shao H. He L. Xiang L. Tang K. Li X. Qi J. (2021). Transparent and UV-absorbing nanocellulose films prepared by directly dissolving microwave liquefied bamboo in TBAA/DMSO co-solvent system. *Ind. Crops Prod.* 171:113899. 10.1016/j.indcrop.2021.113899

[B100] Shen X. Shamshina J. L. Berton P. Gurau G. Rogers R. D. (2016). Hydrogels based on cellulose and chitin: fabrication, properties, and applications. *Green Chem.* 18 53–75. 10.1039/C5GC02396C

[B101] Si Y. Wang L. Wang X. Tang N. Yu J. Ding B. (2017). Ultrahigh−water−content, superelastic, and shape−memory nanofiber−assembled hydrogels exhibiting pressure−responsive conductivity. *Adv. Mater.* 29:1700339. 10.1002/adma.201700339 28417597

[B102] Singh B. Bala R. (2014). Development of hydrogels by radiation induced polymerization for use in slow drug delivery. *Radiat. Phys. Chem.* 103 178–187. 10.1016/j.radphyschem.2014.06.002

[B103] Sui X. Yuan J. Zhou M. Zhang J. Yang H. Yuan W. (2008). Synthesis of cellulose-graft-poly(N,N-dimethylamino-2-ethyl methacrylate) copolymers via homogeneous ATRP and their aggregates in aqueous media. *Biomacromolecules* 9 2615–2620. 10.1021/bm800538d 18774859

[B104] Timofejeva A. D’Este M. Loca D. (2017). Calcium phosphate/polyvinyl alcohol composite hydrogels: a review on the freeze-thawing synthesis approach and applications in regenerative medicine. *Eur. Polym. J.* 95 547–575. 10.1016/j.eurpolymj.2017.08.048

[B105] Tovar-Carrillo K. L. Tagaya M. Kobayashi T. (2013). Bamboo fibers elaborating cellulose hydrogel films for medical applications. *J Mater. Sci. Chem. Eng.* 2013 1–6. 10.4236/msce.2013.17002

[B106] Wang H. Zhang X. Jiang Z. Yu Z. Yu Y. (2016). Isolating nanocellulose fibrills from bamboo parenchymal cells with high intensity ultrasonication. *Holzforschung* 70 401–409. 10.1515/hf-2015-0114

[B107] Wang H. K. Zhang X. X. Jiang Z. H. Li W. J. Yu Y. (2015). A comparison study on the preparation of nanocellulose fibrils from fibers and parenchymal cells in bamboo (*Phyllostachys pubescens*). *Ind. Crops Prod.* 71 80–88. 10.1016/j.indcrop.2015.03.086

[B108] Wang N. Ding E. Cheng R. (2008). Preparation and liquid crystalline properties of spherical cellulose nanocrystals. *Langmuir* 24 5–8. 10.1021/la702923w 18047382

[B109] Wang S. Ren J. Li W. Sun R. Liu S. (2014). Properties of polyvinyl alcohol/xylan composite films with citric acid. *Carbohydr. Polym.* 103 94–99. 10.1016/j.carbpol.2013.12.030 24528705

[B110] Wang W. Xin J. Zhu Y. Zhu C. Tong L. (2016). Effect of vapor-phase glutaraldehyde crosslinking on electrospun starch fibers. *Carbohydr. Polym.* 140 356–361. 10.1016/j.carbpol.2015.12.061 26876862

[B111] Wei G. Zhang J. M. Usuelli M. Zhang X. F. Liu B. Mezzenga R. (2022). Biomass vs inorganic and plastic-based aerogels: structural design, functional tailoring, resource-efficient applications and sustainability analysis. *Prog. Mater. Sci.* 125:68. 10.1016/j.pmatsci.2021.100915

[B112] Xie J. Hse C.-Y. Li C. Shupe T. F. Hu T. Qi J. (2016). Characterization of microwave liquefied bamboo residue and its potential use in the generation of nanofibrillated cellulosic fiber. *ACS Sustainable Chem. Eng.* 4 3477–3485. 10.1021/acssuschemeng.6b00497

[B113] Xie J. L. Hse C. Y. De Hoop C. F. Hu T. X. Qi J. Q. Shupe T. F. (2016). Isolation and characterization of cellulose nanofibers from bamboo using microwave liquefaction combined with chemical treatment and ultrasonication. *Carbohydr. Polym.* 151 725–734. 10.1016/j.carbpol.2016.06.011 27474619

[B114] Xing L. X. Li X. Xing Z. H. Li F. Shen M. W. Wang H. (2020). Silica/gold nanoplatform combined with a thermosensitive gel for imaging-guided interventional therapy in PDX of pancreatic cancer. *Chem. Eng. J.* 382:11. 10.1016/j.cej.2019.122949

[B115] Yang D. Zhong L.-X. Yuan T.-Q. Peng X.-W. Sun R.-C. (2013). Studies on the structural characterization of lignin, hemicelluloses and cellulose fractionated by ionic liquid followed by alkaline extraction from bamboo. *Ind. Crop. Prod.* 43 141–149. 10.1016/j.indcrop.2012.07.024

[B116] Yang G. Z. Kong H. Chen Y. Liu B. Zhu D. Z. Guo L. (2022). Recent advances in the hybridization of cellulose and carbon nanomaterials: interactions, structural design, functional tailoring, and applications. *Carbohydr. Polym.* 279:24. 10.1016/j.carbpol.2021.118947 34980360

[B117] Ye D. Chang C. Zhang L. (2019). High-Strength and tough cellulose hydrogels chemically dual cross-linked by using low- and high-molecular-weight cross-linkers. *Biomacromolecules* 20 1989–1995. 10.1021/acs.biomac.9b00204 30908016

[B118] Yu H. L. Du C. G. Liu H. Z. Wei J. G. Zhou Z. X. Huang Q. L. (2017). Preparation and characterization of bamboo strips impregnation treated by silver-loaded thermo-sensitive nanogels. *BioResources* 12 8390–8401.

[B119] Yu S. Zhang X. Tan G. Tian L. Liu D. Liu Y. (2017). A novel pH-induced thermosensitive hydrogel composed of carboxymethyl chitosan and poloxamer cross-linked by glutaraldehyde for ophthalmic drug delivery. *Carbohydr. Polym.* 155 208–217. 27702506 10.1016/j.carbpol.2016.08.073

[B120] Zhao Y. Zhang X. Wang Y. Wu Z. An J. Lu Z. (2014). In situ cross-linked polysaccharide hydrogel as extracellular matrix mimics for antibiotics delivery. *Carbohydr. Polym.* 105 63–69. 10.1016/j.carbpol.2014.01.068 24708953

[B121] Yuan M. Bi B. Huang J. Zhuo R. Jiang X. (2018). Thermosensitive and photocrosslinkable hydroxypropyl chitin-based hydrogels for biomedical applications. *Carbohydr. Polym.* 192 10–18. 10.1016/j.carbpol.2018.03.031 29691000

[B122] Zainal S. H. Mohd N. H. Suhaili N. Anuar F. H. Lazim A. M. Othaman R. (2021). Preparation of cellulose-based hydrogel: a review. *J. Mater. Res. Technol.* 10 935–952. 10.1016/j.jmrt.2020.12.012

[B123] Zhang H. Zhang F. Wu J. (2013). Physically crosslinked hydrogels from polysaccharides prepared by freeze–thaw technique. *React. Funct. Polym.* 73 923–928. 10.1016/j.reactfunctpolym.2012.12.014

[B124] Zhang J. Song H. Lu L. Zhuang J. Pang C. Liu S. (2012). Microfibrillated cellulose from bamboo pulp and its properties. *Biomass Bioenerg.* 39 78–83. 10.1016/j.biombioe.2010.06.013

[B125] Zheng W. J. Gao J. Wei Z. Zhou J. Chen Y. M. (2015). Facile fabrication of self-healing carboxymethyl cellulose hydrogels. *Eur. Polym. J.* 72 514–522. 10.1016/j.eurpolymj.2015.06.013

[B126] Zhou H. Zhu H. Yang X. Zhang Y. Zhang X. Cui K. (2015). Temperature/pH sensitive cellulose-based hydrogel: synthesis, characterization, loading, and release of model drugs for potential oral drug delivery. *Bioresources* 10 760–771. 10.15376/biores.10.1.760-771

